# Haplotyping the *Vitis* collinear core genome with rhAmpSeq improves marker transferability in a diverse genus

**DOI:** 10.1038/s41467-019-14280-1

**Published:** 2020-01-21

**Authors:** Cheng Zou, Avinash Karn, Bruce Reisch, Allen Nguyen, Yongming Sun, Yun Bao, Michael S. Campbell, Deanna Church, Stephen Williams, Xia Xu, Craig A. Ledbetter, Sagar Patel, Anne Fennell, Jeffrey C. Glaubitz, Matthew Clark, Doreen Ware, Jason P. Londo, Qi Sun, Lance Cadle-Davidson

**Affiliations:** 1 BRC Bioinformatics Facility, Institute of Biotechnology, Cornell University, Ithaca, NY 14853 USA; 2 School of Integrative Plant Science, Cornell AgriTech, Cornell University, Geneva, NY 14456 USA; 3 Integrated DNA Technologies, Redwood City, CA 94063 USA; 4 10x Genomics, Inc., Pleasanton, CA 94566 USA; 5 USDA-ARS, Grape Genetics Research Unit, Geneva, NY 14456 USA; 6 USDA-ARS, Crop Diseases, Pests and Genetics Research, Parlier, CA 93648 USA; 7 Agronomy, Horticulture and Plant Science Department, South Dakota State University, Brookings, SD 57007 USA; 8 Department of Horticultural Science, University of Minnesota, Saint Paul, MN 55108 USA; 9 Cold Spring Harbor Laboratory, Cold Spring Harbor, NY 11724 USA; 10 USDA-ARS, Robert W. Holley Center for Agriculture and Health, Ithaca, NY 14853 USA

**Keywords:** Agricultural genetics, Haplotypes, Natural variation in plants, Plant breeding

## Abstract

Transferable DNA markers are essential for breeding and genetics. Grapevine (*Vitis*) breeders utilize disease resistance alleles from congeneric species ~20 million years divergent, but existing *Vitis* marker platforms have cross-species transfer rates as low as 2%. Here, we apply a marker strategy targeting the inferred *Vitis* core genome. Incorporating seven linked-read de novo assemblies and three existing assemblies, the *Vitis* collinear core genome is estimated to converge at 39.8 Mb (8.67% of the genome). Adding shotgun genome sequences from 40 accessions enables identification of conserved core PCR primer binding sites flanking polymorphic haplotypes with high information content. From these target regions, we develop 2,000 rhAmpSeq markers as a PCR multiplex and validate the panel in four biparental populations spanning the diversity of the *Vitis* genus, showing transferability increases to 91.9%. This marker development strategy should be widely applicable for genetic studies in many taxa, particularly those ~20 million years divergent.

## Abstract (web summary)

Trait introgression requires universal markers, but cross-species transferability of current SNP markers can be as low as 2%. Here, the authors use an AmpSeq haplotype strategy targeting the collinear core genome for marker development and show transferability increases to 91.4% in the *Vitis* genus.

## Introduction

Accelerated breeding is helping to meet the challenge of declining food security in the face of rapid population growth and environmental change. Molecular markers are widely deployed to accelerate crop and livestock breeding programs. These DNA markers are useful for germplasm characterization, marker-assisted selection, marker-assisted introgression, and genomic selection. Over the past four decades, DNA marker systems have evolved from interrogating small numbers of loci and individuals (e.g. Restriction Fragment Length Polymorphisms (RFLPs)^1^, or simple sequence repeats (SSR)^2^) to tens of thousands of loci in large study populations (e.g. fluorescence hybridization-based microarray or next-generation sequencing based genotyping)^3^. However, for breeding applications, single nucleotide polymorphism (SNP) microarrays are constrained by high startup costs and can be affected by ascertainment bias. Alternatively, next-generation sequencing based marker platforms, such as restriction-site associated DNA^4^ and genotyping-by-sequencing (GBS)^5^, suffer from high missing data rates and heterozygote under-calling. This issue has been overcome in grapevine (*Vitis* spp.) through amplicon sequencing (AmpSeq); however, multiplexing of AmpSeq markers is limited to hundreds of loci per PCR reaction due to spurious primer-primer interactions and off-target amplification^6^.

In breeding programs involving highly diverse species and/or genera with rampant structural variation, an important additional concern is marker transferability. For example, Eucalyptus (*Eucalyptus* spp.) breeding efforts draw genes from species that diverged 2 to 5 million years ago (Mya)^7^ while grape breeding can include species that diverged up to 20 Mya^8^. Therefore, universal molecular marker panels are needed that can span the diversity present in broad gene pools. In this study, we use the grape genus (*Vitis*) as a model for the development of a pan-generic marker panel. Cultivated grape (*V. vinifera* subsp. *vinifera*) was domesticated from *V. vinifera* subsp. *sylvestris* around ~6000–8000 years ago^9,10^ and is among the most important horticultural crops in the world^11^. Many grape breeders introgress desirable traits, including abiotic stress tolerance and disease resistance^12–14^, from wild species within the genus. Furthermore, the *Vitis* genus displays a high degree of structural diversity, presenting a challenge for the development of transferable markers.

Multiple factors contribute to the marker transferability problem, including: (1) Null alleles due to local polymorphism. In this case, genetic variability in a PCR primer site or SNP chip probe site causes binding failure, or polymorphism in a restriction enzyme site causes a null allele in a GBS assay. In a study using three SNP chips (BovineSNP50, OvineSNP50, and EquineSNP50) to genotype species that split as long as 50 Mya, the genotyping failure rate increased by 1.5% per million years of divergence^15^. (2) Lack of polymorphism. For example, only 17–33% of the markers on the most widely used SNP genotyping array in maize, the Illumina MaizeSNP50 BeadChip, are polymorphic among European maize inbred lines^16^. Similarly, polymorphism of grape SNP markers drops to as low as 2.3% when applied to different *Vitis* species^17^. Moreover, only 2% of cattle SNPs are polymorphic in water buffalo (diverged ~12 Mya)^18,19^. In a large, multispecies study in animals, Miller et al. showed that polymorphism retention decayed exponentially with divergence time^15^. Only 5% of markers were polymorphic in species that diverged 5 Mya. (3) Genomic structural variation. Many plant species display a high degree of structural variation between individual genomes^20,21^. Markers that fall within the so-called dispensable genome^22^ are less likely to transfer to related species, or even, within the species. Furthermore, they will sometimes be located at different chromosomal positions in different individuals. For example, in *Vitis* we have shown that some markers that tag the flower sex locus in one grape species are located on a different linkage group in other species, even though the flower sex locus itself remains in the same position^6^.

The aim of this study is to develop a low-cost marker system that is transferrable across the entire *Vitis* genus. Our strategy focuses on the colinear core genome and achieves a high level of multiplexing by incorporating RNase H2 enzyme-dependent amplicon sequencing (rhAmpSeq)^23^, which improves the multiplex capacity of AmpSeq via improved amplification specificity and by minimizing spurious primer-primer interactions and off-target amplification. We first identify the *Vitis* core genome based on syntenic whole genome alignment of 10 independent de novo assemblies, consisting of three publicly available genomes (including the *Vitis* reference, *V. vinifera* cv PN40024 genome) and seven linked-read assemblies constructed specifically for this study. Similar to AmpSeq, Illumina sequencing of rhAmpSeq amplicons can capture haplotype allelic series comprised of multiple SNPs and/or Indels per amplicon, resulting in more informative markers than platforms designed for a specific bi-allelic SNP. To help identify highly informative genomic regions and minimize both ascertainment bias and the probability of primer mismatch, we incorporate additional shotgun whole genome sequence data from a *Vitis*-wide diversity panel of 40 accessions. In total, 2000 rhAmpSeq markers are developed, spanning all 19 chromosomes, with an average inter-marker distance of 200 kilobases (kb). The marker panel is validated in four families encompassing much of the genetic diversity in US breeding programs. This strategy generates a highly polymorphic marker set with a low missing data rate across diverse germplasm. The marker set can be used not just for genotyping known alleles, but also for the discovery of novel haplotypes. These short-range novel haplotypes help us to infer the four haplotypes in biparental families directly without any computation estimation. Our strategy should be applicable to many other crop, livestock, and wild species, with or without comprehensive prior genomic resources.

## Results

### De novo genome assemblies of seven *Vitis* accessions

**Fig. 1:**
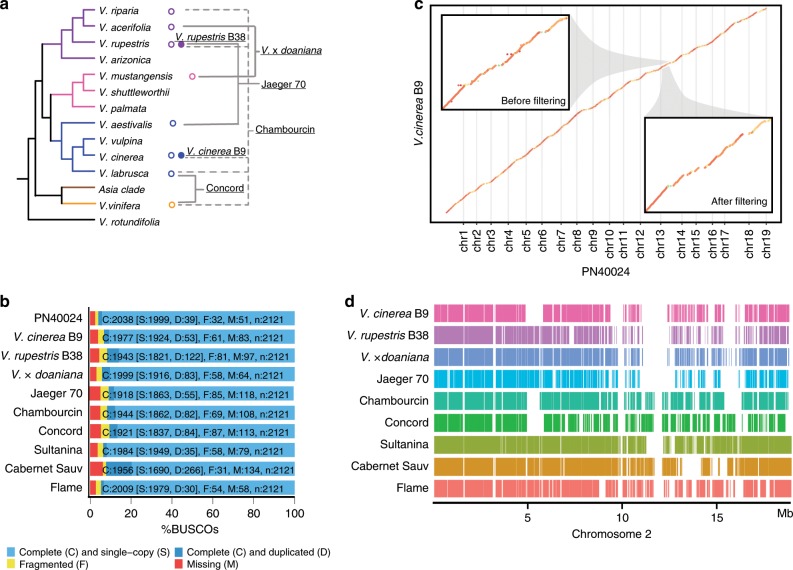
Genome assembly and core genome construction. **a** Simplified illustration of the evolutionary history of the *Vitis* genus and the genomes included in this study. To enrich diversity in the primer design panel, interspecific hybrid grapes with diverse genetic backgrounds were sequenced. Open circle denote species represented by a sequenced hybrid accession; Closed circle denote one non-hybrid accession from this species was sequenced. Dotted lines denote more than two taxa were included in the accession. **b** BUSCO scores for the ten genomes examined in this study. **c** Synteny of the raw 10x assembly with the reference. The upper left panel is the raw pairwise genome alignment of a region on chromosome 14 constructed by minimap2; the lower right panel is filtered alignment of the same region requiring a syntenic one-to-one match. **d** Coverage of chromosome 2 across the nine genomes. Each line indicates a region that can be aligned to the reference.

For accurate definition of the core genome, genome assemblies are required for a representative panel that spans the genomic structural diversity present in the target taxon. The de novo assembly panel in this study included two accessions from wild *Vitis* species, two accessions of wild-wild interspecific hybrids, two accessions of interspecific hybrid grape cultivars (crossing of wild and domesticated species), and four accessions of widely-cultivated modern cultivars of *V. vinifera* subsp. *vinifera* including the reference genome PN40024 (Supplementary Table 1, Fig. 1a). The genomes of Sultanina (a seedless table grape)^24^ and Cabernet Sauvignon^25^ were obtained from the public database. In addition, we de novo assembled seven genomes using linked reads, with total raw sequencing read depths ranging from 39-fold to 66-fold. After assembly with the Supernova Assembler (version 2.0.1), contig N50s ranged from 43.9 to 56.86 kb, and the scaffold N50s ranged from 278 kb to 2.1 megabase pair (Mbp). For all nine genomes, more than 90% of the BUSCO genes were represented in full length, indicating good coverage of the gene space (Fig. 1b). Supernova 2.0.1 produces diploid assemblies comprised of two locally phased pseudo-haplotypes. We found that these two phased pseudo-haplotypes only contain one heterozygous site every 371–406 base pairs, which might be due to the under-calling after misassembly of one haplotype. Therefore, only one pseudohap1 assembly was used to represent each genome in the downstream analyses. The molecular length, effective depth, and assembly statistics for each genome are shown in Supplementary Table 1.

### Construction of genus-wide core genome

To construct the *Vitis* core genome, the nine assemblies were aligned to the grape reference genome (PN40024 version 12X.v2^26^). As 41.4% of the grape genome is composed of transposable elements^11^, we masked the repetitive genomic regions prior to alignment by kmer frequency. A high degree of collinearity between each assembly and the reference was observed, validating the assembly qualities in general. To further maximize one-to-one correspondence, the pairwise syntenic alignments were smoothed by collapsing small, local tandem duplicates, present either in the PN40024 reference or the assembly (Fig. 1c, Supplementary Fig. 1). The total length of one-to-one matched blocks after smoothing ranged from 160 to 226 Mbp and depended on genetic distance to *V. vinifera*. After the kmer-based repeat masking, 88% of the coding sequences were retained on the reference genome PN40024. For the other genomes aligned with PN40024, before smoothing 64 to 77% of the reference coding sequences were retained, and 55 to 39% were retained after smoothing (Supplementary Table 1). Core genomic regions were distributed across each chromosome, except for gaps that represent either structural variation (the dispensable genome) or mis-assembly. As expected, the genomes of the wild species display more structural variation relative to the reference genome than those of domesticated cultivars. For example, on average, only 37% of the reference genome was collinear with wild species versus 54% with cultivars (Supplementary Table 1). An illustration of collinearity with chromosome 2 of each assembly is presented in Fig. 1d. The total length of the core genome decays exponentially as more assemblies are included in the core genome (Supplementary Fig. 2). By fitting an exponential decay function, the inferred core genome size is predicted to plateau at 39.8 Mbp with 17 assemblies in the model.

We define the coverage of the core genome presence as the number of times a reference PN40024 chromosomal region aligned with another genome assembly in a collinear, syntenic fashion. About 10% of the reference genome was present in all nine genome assemblies—we define these regions as the grape core genome. Sixty-four percent of the inferred core genome lies within gene regions. We consider these 9386 genes in the collinear core genome to be core genes, and they are significantly enriched in cellular metabolic processes, RNA binding function, and membrane localization (Supplementary Fig. 3). Certain genome features are more prevalent in the core genome (Supplementary Fig. 4). For example, gene density is higher in the core genome versus the remaining genome (*ρ* = −0.75, *P* < 1E-13 in two-sided Spearman’s correlation test) and transposable elements (TEs) are depleted (*ρ* = 0.76, *P* < 1E-13 in two-sided Spearman’s correlation test), in particular the most abundant TE family, Gypsy.

### Marker design and statistics

**Fig. 2:**
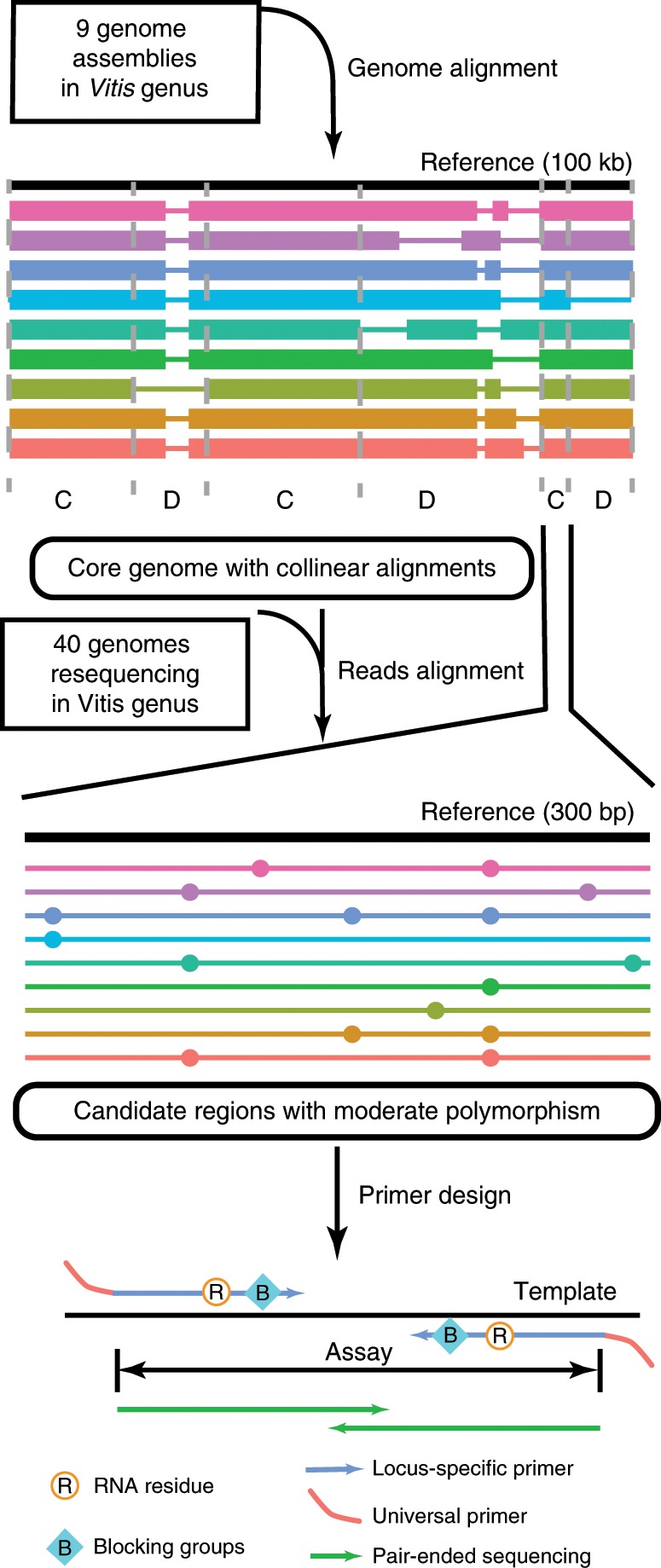
Marker design pipeline based on genus-wide core genome and polymorphism. Colored blocks, alignment between the query genome and the reference genome. C, core genome; D, dispensable genome. Colored dots denote genomic variants. In the rhPCR, an RNA residue and several bases of blocking DNA are added to the allele-specific primer. The RNA residue together with the blocking DNA can only be cleaved by the RNase H2 enzyme when the match between the target and the primer is perfect. After the RNA residue and the blocking DNA are removed from the 3′ end, the extension reaction will continue.

To develop markers universally effective in diverse germplasm, rhAmpSeq markers were designed to target only the core *Vitis* genome, with multiple additional attributes taken into consideration (Fig. 2). First, to decrease off-target amplification, primer binding sites had to be unique and had to avoid sequence variation. Second, the polymorphism level of PCR products had to be moderate. Elevated polymorphism is both advantageous and disadvantageous in marker design. A higher level of polymorphism manifests as multiallelic haplotype markers, which are more informative than biallelic ones; however, it also increases the risk of null alleles or off-target amplification from its paralogs in the genome. In our genotyping pipeline, instead of calling multiple independent SNPs separately, the entire amplicon of 200–300 bp is used as a haplotype allele tag. To extensively characterize genus level polymorphism in these targeted regions, we called variants based on grape whole genome sequencing data from two sources: (1) 47 *Vitis* accessions with at least three-fold sequencing depth retrieved from the NCBI SRA, and (2) seven *Vitis* accessions shotgun sequenced specifically for this study (these are different from the seven de novo assembled accessions) (Supplementary Table 2). Principal component analysis of the resulting genotypes indicated that the wild species are substantially more genetically diverse than the cultivated lines (Supplementary Fig. 5). To balance the composition of wild species and cultivars in this diversity panel, we randomly selected twenty accessions from each group. Across the 40 accessions, SNP density in the core genome was 0.032 (i.e., 32 SNPs per kilobase), and was very similar in the core genes (0.031) (Supplementary Fig. 6). To balance primer transferability against information content, we focused on moderately polymorphic regions by discarding loci outside the 25th and 75th percentiles. As expected, the missing genotype rate in the *Vitis* diversity panel decreases as an exponential decay as more assembled genomes are included in the core genome construction (Supplementary Fig. 7). The final consideration for marker design was physical distribution across the genome. Initially, candidate regions were randomly chosen to obtain one marker per ~200 kb of reference genome. Subsequently, to improve efficiency in gene mapping, we included more gene-rich regions, where the recombination rate is typically elevated^27^. Successful primer designs were obtained for 99.6% of the candidate regions, with amplicon size ranging from 270 to 330 bp (Supplementary Fig. 8). Of these, 98% were predicted to be multiplex-competent in a single reaction. In total, 2000 rhAmpSeq markers were designed and synthesized (Supplementary Data 1).

### Marker validation in four grapevine families

The 2000 rhAmpSeq marker panel was then evaluated in the four grapevine breeding families representing a wide range of genetic diversity in US breeding programs, including wine grapes, table grapes, wild species, and interspecific hybrids; hereafter, each family is referred to using the two-letter initials preceding its description: (1) HC: “Horizon” × *V. cinerea* B9 (PI588154), a complex F_1_ family maintained at Cornell University in Geneva, New York and including *V. vinifera, V. cinerea, V. aestivalis* var*. lincecumii*, and *V. rupestris*^6,28^. (2) MN: MN1264 × MN1246, a complex F_1_ family maintained at the University of Minnesota Horticultural Research Center in St. Paul, Minnesota and including *V. vinifera, V. riparia, V. rupestris, V. labrusca, V. aestivalis*, and *V. cinerea*^6,29^. (3) RS: *V. riparia* 37 (PI588259) × “Seyval blanc”, an F_2_ family maintained at South Dakota State University in Brookings, South Dakota and including *V. riparia, V. rupestris*, and *V. aestivalis*^30^. (4) BC: B37-28 × C56-11, a modified backcross (mBC_1_) family maintained at USDA-ARS in Parlier, California and including *V. vinifera* and *V. aestivalis*^31^.

**Fig. 3:**
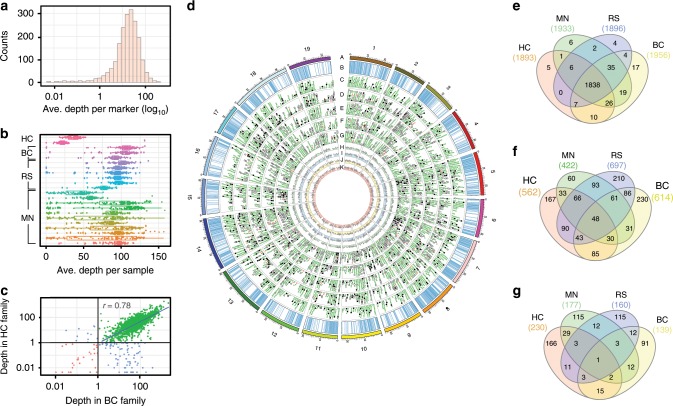
Performance of 2,000 rhAmpSeq markers in four families. **a** Distribution of average depth of markers on a log10 scale. **b** Average depth per sample in all plates of four families. Each point represents one sample in the plate. The bound box represents the 25th or 75th percentiles and the whiskers extends to 1.5 times of the interquartile range. **c** Correlation of read depth in two families. The Pearson correlation coefficient was calculated for the markers with average depth larger than one in both families. **d** Circos plot of marker performance in four families and the metapopulation. **a** denotes Chromosomes; **b** denotes physical locations of rhAmpSeq core genome markers; **c**–**g** denote rhAmpSeq markers that failed to return data (red triangle), distorted (black square), monomorphic (white square) and on linkage map (green circle) for consensus genetic map, HC, MN, BC and RS families; **h**–**k** denote read depth across for each marker in HC, MN, BC, RS families, respectively. **e** Venn diagram of the markers with average read coverage greater than 1 across the four families. **f** Venn diagram of the monomorphic markers in four families. **g** Venn diagram of the distorted markers in four families.

Firstly, to examine amplification and sequencing bias in the rhPCR, we calculated the average read depth for each marker (Fig. 3a). After log (base 10) transformation, sequencing depth was nearly normal in distribution, and 90% of markers ranged from 1- to 100-fold in depth, indicating that the amplification was efficient for most markers, and depth was sufficient for genotyping. Secondly, we checked the reproducibility of the rhAmpSeq platform in generating similar data quantities among 96-well plates of samples, using different DNA extraction protocols and Illumina sequencers. The average sequencing depth per sample was greater than ten for all 96-well plates (Fig. 3b). The number of individuals with depth <10 in the MN family was significantly higher than in other families (*χ*^2^, two-tailed chi-squared test, *P* < 1E-6), as was the variance of read depth, likely due to different DNA extraction protocols. The DNA of the MN family was extracted using an automated magnetic bead pipeline, while the other families were manually extracted using commercial kits that included a column-based purification step. The HC family had less depth than the others, as it was sequenced on a MiSeq, which has a lower output than the NextSeq500 used for the other families. Thirdly, we examined the correlation of marker sequencing depth between two families. After excluding markers with average depth less than one, the Pearson correlation coefficient (*r*) was 0.78, indicating that sequencing depth is mainly influenced by the composition of the probe and the target sequence, and less so by the genetic background (Fig. 3c).

The performance of each marker is illustrated in a circos plot (Fig. 3d) and summarized in Table 1. Despite no previous testing or troubleshooting of amplification conditions, very few markers displayed null alleles, and 91.9% (1838) of the markers had a mean read depth of at least one in all four families (Fig. 3e). In addition, markers classified as missing, monomorphic, or segregation-distorted were distinct for each family (Fig. 3e–g), and 97.1% of the markers amplified in at least one of the families. As expected, more markers were monomorphic in the F_2_ RS family (697) and in the modified backcross BC family (614) with narrow genetic distances shown in Supplementary Fig. 9, than in the wider, multi-species F_1_ crosses HC (562) and MN (422). The rhAmpSeq markers had a mean of 5.7 alleles per marker across seven genotyped parental accessions (Supplementary Fig. 10).

**Table 1:** Summary statistics of the genetic maps of each family and the consensus genetic map.

	HC	MN	RS	BC	Consensus Map
Number of vines	157	1007	504	260	600
Cross type	F_1_	F_1_	F_2_	F_1_	–
rhAmpSeq core genome markers	2000	2000	2000	2000	2000
Markers in linkage groups	1153	1387	1113	1222	1661
Markers that failed to return data	55	14	30	25	31
Monomorphic markers^a^	562(34)	422(48)	697(111)	614(42)	111
Distorted markers^a^	230(47)	187(93)	160(11)	139(50)	228
Male genetic map size (cM)	2224.2	1760.8	1415.8	1787.7	–
Female genetic map size (cM)	1333.4	1519.0	1569.5	1502.9	–
Sex-averaged genetic map size (cM)	–	–	–	–	1198.1
Male genome-wide recombination rate (cM/Mbp)	4.9	3.8	3.1	3.9	–
Female genome-wide recombination rate (cM/Mbp)	2.9	3.3	3.4	3.3	–
Sex-averaged genome-wide recombination rate (cM/Mbp)	–	–	–	–	2.61
Male genome-wide correlation of the genetic and physical map (*r*)	0.79	0.93	0.79	0.86	–
Female genome-wide correlation of the genetic and physical map (*r*)	0.86	0.90	0.80	0.86	–
Sex-averaged genome-wide correlation of the genetic and physical map (*r*)	–	–	–	–	0.95
Genome^b^ coverage (%)	95.9%	98.2%	96.9%	96.4%	98.6%

Abbreviation: HC, Horizon × *V. cinerea* B9; MN, MN 1264 × MN 1246; RS, *V. riparia* 37 × Seyval; BC B37-28 × C56-11

^a^The number in the parentheses indicates the number of markers with a null allele segregating according to Mendelian expectations

^b^ Relative to 12X.v2 version of the PN40024 reference genome

### Core-genome mapping indicates possible genome divergence

**Fig. 4:**
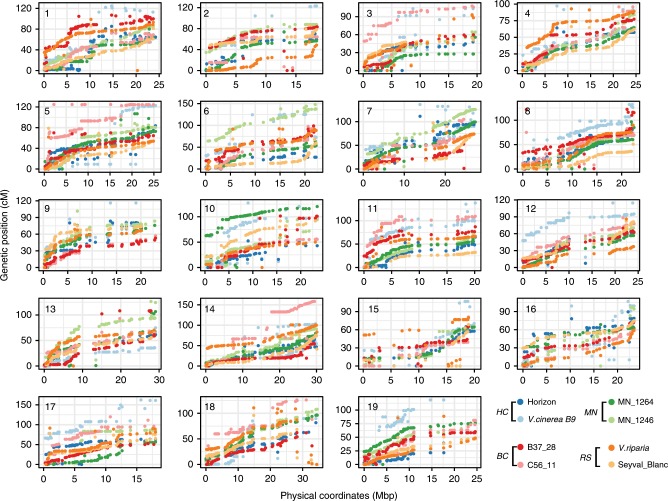
Relationship between the genetic and physical maps for the nineteen chromosomes of the parents in four families. Genetic distance: cM; Physical maps: Mbp. The genetic distances of the markers were derived from the genetic map developed for each family and physical distances are from version 12X.2 of the PN40024 reference genome.

We constructed parent-specific genetic maps based on the segregating markers in each of the four families. The total genetic distances ranged from 1333.4 to 2224.2 cM (Table 1). The average Pearson’s correlation (*r*) between physical and genetic positions ranged from 0.79 to 0.93 genome-wide, and the genetic maps covered 95.9–98.2% of the reference genome (Supplementary Data 2). In general, the parental genetic maps were highly similar among parents, but there were some parent- or family-specific anomalies. For example, the distal 30.1% of chromosome 19 was monomorphic (non-segregating) for both parents of HC. Other regions failed to recombine in a specific parent, with the most extreme case being no recombination over the distal 64.4% of chromosome 17 in the female parental map of the MN family (Fig. 4). Parent-specific repression of recombination indicates candidate regions of structural variation in the genome revealed by the core markers, for further exploration.

We analyzed markers excluded from the linkage maps to determine whether they perform poorly in all families. Almost all excluded markers were monomorphic or displayed segregation distortion (Table 1). Most monomorphic markers or distorted markers (47.7% or 77.3%, respectively) were specific to one family, and only 18.6% of the monomorphic markers and 5.5% of the distorted markers were in problematic three or four families (Fig. 3). We found that distorted markers were enriched in minor allele frequency surrounding 0.33, most likely due to the hemizygosity, or null alleles, in one of the parental genomes. In a recently published genome of a highly heterozygous cultivated grape, 14.2% of the genes were hemizygous^21^. We tested if the distorted markers segregated in a Mendelian pattern when allowing hemizygosity among the alleles. We found that up to 52% of distorted makers and up to 15% of monoallelic markers followed Mendelian laws of segregation if assuming one or two alleles were hemizygous in the parents (Table 1). While missing one copy of the allele could have a biological basis (deletion in the genome), it could also be attributed to mismatches in the primers resulting in failed PCR amplification. By inferring the genetic variances at the primer regions using the whole genome shotgun sequences of four parents, we found that 83% of the markers with a potential hemizygous allele had at least one mismatch in the primers, which is two times higher than markers with no hemizygous allele. Thus, our primer design pipeline successfully targeted the core genome, avoiding biologically hemizygous sites, but returned null alleles in about 5% of markers per parent. Nevertheless, we were able to place 83% of the 2000 markers in this panel on a consensus genetic map using a joint-linkage mapping population of 600 vines (150 vines from each family), with an average Pearson’s correlation (*r*) between physical and genetic positions of 0.95 across all chromosomes and 98.6% coverage of the physical genome (Supplementary Data 1 and Supplementary Fig. 11). This high genetic mapping rate indicates that most markers should behave as true Mendelian markers in most *Vitis* taxa.

### A transferable flower sex marker

**Fig. 5:**
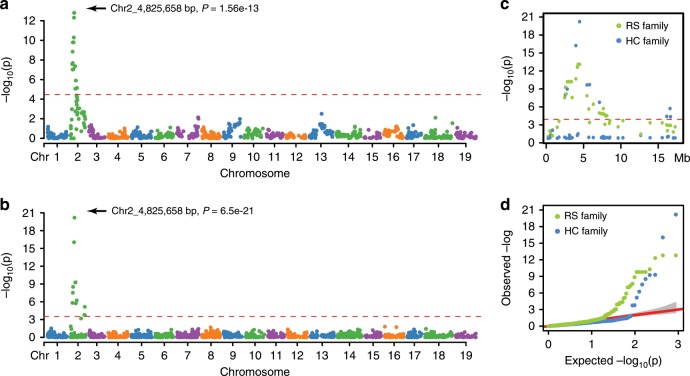
Association mapping of flower sex loci in the RS and HC families. **a**, **b** Manhattan plot of GWAS results for flower sex in the RS and HC families, respectively. Bonferroni threshold at 4.5 (*α* = 0.05) is shown in dotted red line. **c** The *p*-values on chromosome 2 only. **d** QQ-plot for the flower sex GWAS analysis in the RS and HC families. Source data are provided as a Source Data file.

In the *Vitis* genus, all of the wild species are dioecious while the domesticated grapevine *V. v*. ssp*. vinifera* is hermaphroditic^32^. The region around 5Mbp on chromosome 2 has been identified in several linkage mapping and population genetic studies, and the boundary of the sex locus and the sex determining genes were proposed but are still under debate^32–36^. Here, we used the sex locus to assess the performance of our rhAmpSeq markers in genetic mapping. A total of 1712 and 1784 post-imputed, filtered markers were analyzed for association with the flower sex trait measured in 146 and 106 vines from the HC and RS families, respectively. After Bonferroni correction for multiple comparisons, 13 and 17 markers, respectively, were significantly associated with flower sex. Marker *chr2_4825658* (chromosome 2 at position 4,825,658 bp) was the most significant marker in both the HC (*P* *=* 6.5E-21, male (M) allele dominant over female (f)) and RS (*P* = 1.56E-13, hermaphrodite (H) allele dominant over female (f)) families, and was concordant with flower sex phenotype for 143/146 HC progeny (97.9%) and for 100/102 RS progeny (95.3%) (Fig. 5). In our previous study of the sex locus using genotyping-by-sequencing markers, the distinct genetic markers associated with flower sex inconsistently spanned a 1 Mb region in different mapping families^6^. In contrast, here the same marker was most significant in both HH × Mf and Hf-selfed families, which emphasizes the transferability of these core genome markers.

## Discussion

A set of universal genetic markers that work for related taxa is desired in many genetic studies. In marker-assisted breeding, universal markers can be used in crosses between distant relatives to generate heterosis or introgress useful alleles^37–39^. In molecular ecology and evolutionary studies, universal markers allow comparison of genetic characters among related species^40,41^. In genera or families containing many economically important species, transferable, universal markers can decrease the time and effort required for marker development^42,43^. While the transferability for low-throughput microsatellite (SSR) markers is relatively good, ranging from 27% to 77% in the different taxa of plants and animals^44^, the transferability of high-throughput SNP genetic markers have been as low as 2%^17,45^.

In this study, we developed and validated a pipeline for designing universal markers that work across the diverse *Vitis* genus, which were diverged 20 Mya. Using the rhAmpSeq targeted sequencing platform, 93% of markers returned data for all four families tested, and around 70% of markers were polymorphic in every family. All parental genetic maps were highly correlated with physical position in the PN40024 reference genome (*r* = 0.86 to 0.95). Although 10 to 20% of the markers in each family deviated from expected Mendelian segregation ratios, these markers were family-specific and were clustered on particular chromosomes. The vast majority of markers were informative for consensus genetic map construction, indicating high marker transferability. Furthermore, in two families where the sex trait was analyzed, the same marker explained the most phenotypic variation and was the most significantly associated. This result suggests that not only are random markers transferable, but functional markers are also transferable. Thus, it appears that markers designed to target a genus-wide core genome are transferable in all key aspects, including amplification, polymorphism, segregation, and marker-trait association.

The design of transferable markers depended on the construction of a genus-wide core genome comprised of collinear, syntenic blocks. Previously, markers designed from shotgun resequencing had limited transferability because only local genetic variation could be assessed, and large and complex structural variation was often overlooked. Any long collinear block conserved within a structurally diverse taxon is suggestive of strong selection against structural variation within the block, and markers designed within such blocks are more likely to consistently occur in different species with consistent segregation patterns. For this study, linked-read scaffolding of de novo assembly enabled the identification of collinear blocks at a relatively inexpensive price point (about $3000 per 475 Mbp genome). By using genus-wide sequence data to design primers targeting conserved sequences flanking regions of moderate polymorphism in the inferred core genome, we obtained markers that reliably returned informative data in most cases.

Previously, we found that the AmpSeq genotyping platform outperforms GBS for highly diverse and heterozygous species, due to reduced missing data, increased coverage and increased accuracy at heterozygote sites, as well as elevated transferability among species^6^. In contrast to SNP arrays or Kompetitive allele-specific PCR (KASP), which typically target two alleles per marker, or site, the AmpSeq genotyping platform allows identification of numerous alleles as short haploblocks because the entire amplified target (typically 200–250 bp) is sequenced via NGS. The rhAmpSeq markers developed in this study had a mean of 5.7 alleles per marker across seven genotyped parental accessions (Supplementary Fig. 10). Compared to biallelic SNPs, these multiallelic haplotype markers simplify phasing along the chromosome and provide more information about ancestry. The high information content, even coverage, and unbiased sequencing of rhAmpSeq amplicons make this platform applicable for population genetics and ecology studies. Relative to AmpSeq, the rhAmpSeq technology simply adds an RNA base and blocker DNA at the 3′ end of each primer. When the match is perfect between the primers and template, this RNA-base and blocker are cleaved by RNase H2 enzyme^46^. This step increases the genotyping specificity and increases the multiplexing capacity up to 5000 markers per reaction^46^.

Here we developed a strategy for genus-wide haplotype marker design considering the syntenic core genome and genus-wide polymorphism. In combination with the rhAmpSeq platform, this genotyping pipeline can be easily adapted for other taxa for ecological and evolutionary studies, QTL mapping, GWAS, and molecular breeding. The costs for rhAmpSeq highly depend upon the number of markers and samples, as primer and reagent prices are subject to scale, and sequencing costs are reduced by greater sample multiplexing. Given existing DNA and perfect efficiency, a large project could generate sequencing reads for 2000 rhAmpSeq markers for as little as $4 per sample plus rhAmpSeq reagent costs. With the recent availability of inexpensive linked-read sequencing, this cost-effective strategy will be particularly useful for crops (or other eukaryotes) with poor genomic resources where it will now be possible to develop core genomes, and especially for genera or families (e.g., Poaceae or Rosaceae) that would benefit from a universal set of highly transferable markers.

## Methods

### DNA processing and genome assembly

For de novo sequenced vines, rapidly expanding leaves about two-centimeter (cm) long were collected for six samples from vineyards in Geneva, New York (Supplementary Table 1). High-molecular-weight (HMW) genomic DNA (gDNA) was isolated using a CTAB protocol modified from Japelaghi et al. and Haley et al.^47,48^. The genomic DNA was quantified with Quant-iT kits using a Qubit fluorometer (Thermo Fisher Scientific), quality checked with a Thermo Nanodrop™ 2000 Spectrophotometer (Thermo Fisher Scientific), and sized via Pulsed Field Gel Electrophoresis. The bulk of the gDNA smear was required to be >60 kb before further processing and was typically centered on 100 kb. For seven of the genomes listed in Supplementary Table 1, HMW DNA was shipped to 10X Genomics (10X Genomics Inc., Pleasanton, CA, USA) for preparation and sequencing of libraries following their standard protocols (10X Genomics; Pleasanton, CA). Each 10X Genomics library was sequenced to between 32- and 66-fold coverage on an Illumina NovaSeq sequencer to generate linked-reads with a mean read length of 139.5 bp after trimming. The whole genome sequencing (WGS) library preparation and sequencing was performed at 10X Genomics. The linked-read data were assembled using Supernova v.2.0.1 assembler^49^ with default settings. The weighted mean molecule size was estimated by the Supernova software as 63.18 kb and mean read coverage as ~68 fold. The BUSCO score was calculated based on lineage-specific sets of Eudicotyledons odb10 with genome model (BUSCO: version 3.0.2, AUGUSTUS: Version 3.3).

### Syntenic core-genome construction

Repetitive regions in both the reference genome and the assemblies were masked using a kmer frequency-based approach with BBduk, part of the BBTools package (version 35.50)^50^. Sequences with a kmer (*K* = 31) frequency larger than two were replaced with Ns. The masked assembly was aligned to the reference genome, PN40024 (version 12X.v2)^26^, using Minimap2 with parameter presets tuned for cross-species alignment, denoted as “asm10” in the manual^51^. By the definition in minimap2, each alignment has a global alignment score larger than 400 (defined by -z) and with >90% identity with the reference (defined by -x asm10).The results were transformed to a bed-like format for chaining and identifying one-to-one matches of chains with at least three alignments derived from minimap2 using quota_alignment (https://github.com/tanghaibao/quota-alignment)^52^. The total length of each collinear core-genome alignment that is larger than 10 kb were kept in the downstream analysis. In this study, we defined the core genome as the chromosomal regions of reference PN40024 that had collinear, syntenic alignment with all other genome assemblies. The core genome coverage, gene density, transposable element (TE) density, and other genome features were calculated with window size of 1 Mb using BEDTools (v2.27.1). The correlation between each pair of genome features was calculated using Spearman’s rank correlation coefficient in R software (Version 3.5.0).

### Genus-wide variant calling

A total of 47 *Vitis* accessions with 3- to 93-fold paired-end Illumina sequencing data were downloaded from the National Center for Biotechnology Information (NCBI) Sequence Read Archive (SRA). If one accession had more than 20-fold read coverage, we randomly down-sampled to 20-fold read coverage for variant calling to avoid bias in variant calling from high-depth samples and to save processing time. We also sequenced seven accessions with 8- to 160-fold paired-end sequencing using the Illumina HiSeq 2500 platform. Reads were processed and variants were called based on PN40024 (version 12X.v2)^26^ using the Sentieon DNA Software Package (version, Golden helix)^53^ with default settings. This Sentieon package is a speed-up software that rebuilt the Genome Analysis Toolkit HaplotypeCaller and returns the same result as GATK 3.3. Principal component analyses (PCA) was conducted using R/Bioconductor Package SNPRelate^54^. To avoid the strong influence of clustered SNPs in the PCA analysis, the SNPs were filtered using LD-based pruning algorithm implemented in SNPRelate with LD threshold 0.2. We arbitrarily selected 20 *V. vinifera* samples and 20 non-*vinifera* samples.

### Marker design pipeline

The VARIANT CALLING FORmat (VCF) file generated from the genus-wide variants calling was loaded into R software. For each aligned region in the core-genome, the length, diversity and missing rate was calculated. The regions that were shorter than 200 bp, with diversity larger than 7% or smaller than 2%, or with average missing rate larger than 50% were removed. These steps were conducted in R using the bioconductor (version 3.8) package VariantAnnotation^55^. The candidate regions were then picked to ensure one marker per 200 kb. If no qualified candidate region could be found in a 1 Mbp window, we included the regions that had highest coverage in the core genome construction. To achieve a better representation of gene rich regions with high recombination rates, we included more candidate regions with high gene density. A total of 2500 candidate regions were sent to Integrated DNA Technologies, Inc. (IDT, Coralville, IA, USA) for primer design and pooling compatibility test. Primers could be designed for 99.6% of the regions, and 98.1% of them were pooling compatible in a single PCR amplification reaction. A total of 2000 rhAmpSeq primer pair assays were synthesized by IDT.

### rhAmpSeq sequencing and genotyping

The DNA of the MN family was extracted by Intertek AgriTech (Sweden) using an automated magnetic bead pipeline with sbeadex kit provided by LGC (Teddington, United Kingdom). The DNA of other families was extracted using QIAGEN DNeasy 96 Plant Kits manually. We modified the protocol to include 3% w/v PVP40 to the lysis buffer prior to extractions to remove PCR inhibitors. rhAmpSeq amplification enrichment using the 2000 marker panel was conducted following manufacturer’s protocol. Briefly, the first PCR used 14 cycles with annealing temperature at 61 °C for each sample. The PCR products were diluted 1:20 and indexed with IDT indexing primers using 24 cycles with an annealing temperature at 60 °C. The indexed PCR products were pooled, cleaned with Agencourt AMPure beads, quantified, and sequenced on an Illumina (Illumina, San Diego, CA, USA) MiSeq (2 × 150 bp) or NextSeq (2 × 150 bp) sequencer. rhAmpSeq sequencing data for all the four genetic mapping families were initially analyzed using the Perl script analyze_amplicon.pl (https://github.com/avinashkarn/analyze_amplicon/blob/master/analyze_amplicon.pl), and later re-analyzed with an upgraded pipeline optimized for rhAmpSeq data analysis (https://bitbucket.org/cornell_bioinformatics/amplicon). The pipelines de-multiplex the sequencing reads based on PCR primer sequence, obtain haplotype variants for each marker across all vines in the four families, and generate a sample to haplotype allele matrix. Both PCR errors and sequencing errors produce false haplotype alleles. To correct these errors, haplotype variants caused by genotyping errors were collapsed by filtering out nucleotide sites within homo-polymer repeats or deviating from expected Mendelian segregation ratios of bi-parental families. In rare cases where a haplotype marker had no sites segregating at the expected ratio, the site with the biggest minor allele frequency was used to represent the haplotype allele. Monomorphic markers and markers with greater than seventy five percent missing data in ‘hapgeno’ file were manually removed from the further analysis. Finally, using a custom Perl script, haplotype_to_VCF.pl (https://github.com/avinashkarn/analyze_amplicon/blob/master/haplotype_to_VCF.pl), the four most frequent haplotype alleles for each marker (within a family) in the *hapgeno* file were converted to a VCF file, where each haplotype allele of a marker was converted to a pseudo A, C, G or T allele, for further marker validation analyses that are discussed hereafter.

### Imputation and filtering

The raw converted VCF files for each grapevine family were imported in TASSEL (Trait Analysis by association, Evolution and Linkage) 5.2.51 software^56^ and the genotypes were imputed using the LD-kNNi imputation plugin also known as LinkImpute (v1.1.4)^57^ using the default parameters (High LD Sites = 30, Number of nearest neighbors = 10, and Max distance between site to find LD = 10,000,000). Post-imputation, vines with >90% missing data were removed from the analysis.

### Multidimensional scaling for quality control analysis

Post-imputed and filtered markers were used to calculate a genome-wide pairwise identity-by-state (IBS) distance matrix for each family in TASSEL software using 1–IBS followed by Multidimensional scaling (MDS) analysis^56^. The first three principal coordinates in the multidimensional scaling of each family were graphically depicted using the R statistical software. The MDS analysis predicted the hidden population structure by separating four clusters of progeny vines relative to their corresponding parents and grandparents, and indicated vines that were self-pollinated, outcrosses, or mislabeled, which were removed from linkage mapping and GWAS analysis.

### Construction of genetic maps

Genetic maps were constructed in Lep-MAP3 v.0.2^58^ (LM3) using the VCF file of post-imputed and filtered markers as well as pedigree information for each family. The following LM3 modules and steps were used to construct the genetic maps: (1) *ParentCall2* module of LM3 was used to call parental genotypes; (2) the resulting output was filtered by using *Filtering2* module (parameter *dataTolerance* = 1.00E-3 for F_1_ and mBC_1_ families and 1.00E-10 for the F_2_ family), and the markers were filtered out based on a two-sided *χ*^2^ (chi-squared) test (testing if the allele ratio is significantly deviated from the expected mendelian ratio, at the above tolerance thresholds) or monomorphism; (3) *SeparateChromosomes2* module was used to identify linkage groups using logarithm of odds (LOD) score limit ranging from none to 20 for the individual family (Table 1); (4) Finally, *OrderMarkers2* module was used to compute the parental genetic distances (sex specific for the F_1_ and mBC_1_ families and sex averaged for the F_2_ family) of the markers in the linkage groups using 20 iterations per group. Correlation plots of genetic and physical distances of individual markers per chromosome in each family were plotted to evaluate the consistency of the maps, genome organization and structural variation.

Further, a consensus genetic map was constructed in LM3, where 150 progeny vines from each family were randomly chosen, and their genotypes were merged into a single VCF file in TASSEL using ‘Merge Genotype Table’ plugin. The merged VCF file and pedigree information containing all four families were used in LM3 as an input to construct the sex averaged consensus genetic map as described above.

### Genome-wide association study of flower sex

The association between a well-characterized trait (flower sex) and genotypes was used to further evaluate rhAmpSeq marker transferability. Specifically, a genome-wide association study (GWAS) was conducted to map the flower sex locus in HC (F_1_) and RS (F_2_). The male allele (M) is dominant to hermaphroditic (H), which is dominant to female (f), that is, M > H > f. HC represents a cross of homozygous hermaphroditic flowers (HH) emasculated and pollinated with pollen from a male (Mf) vine, and should segregate 1 male (MH): 1 hermaphrodite (Hf). RS represents self-pollination of heterozygous hermaphroditic flowers (Hf) and should segregate three hermaphrodite (HH/Hf/Hf): one female (ff).

GWAS was conducted in TASSEL on post-imputed and filtered markers in the two families with their respective flower sex phenotypic values and phenotypes using a mixed linear model (MLM). As covariates, the first two principal components of MDS analysis was were used to correct for population structure (P), and the kinship matrix (K), the proportion of alleles shared between each pair of vines was used to correct for familial relatedness as covariates. The Eq. (1) for MLM (P + K) model was:

1 $${\mathrm{y}} = X{\upalpha} + P{\upbeta} + K{\mathrm{u}} + \epsilon$$

where, y is a vector of a phenotypic data, *α* is the fixed effects related to the marker, β is a vector of the fixed effects related to the population structure, u is a vector of the random effects related to the relatedness among the vines, and ϵ is a vector of the residual effects. *X* is the genotypes of the marker, *P* is the matrix of principle components, *K* is the centered identity by state (IBS) kinship matrix.

Phenotypic variability explained by each significant marker was estimated by *R*^*2*^ values generated in MLM statistics output from TASSEL software^56^. Further, the Bonferroni-corrected threshold was determined for each association analysis using 1/*N* (*α* = 0.05), where *N* is the number of markers tested, and quantile-quantile (QQ) plots were utilized to examine model fitness for the flower sex trait in each family.

### Reporting summary

Further information on research design is available in the Nature Research Reporting Summary linked to this article.

## Supplementary information

Supplementary Information

Reporting Summary

Description of Additional Supplementary Files

Supplementary dataset 1

Supplementary dataset 2

## Data Availability

Data supporting the findings of this work are available within the paper and its Supplementary Information files. A reporting summary for this Article is available as a Supplementary Information file. The datasets generated and analyzed during the current study are available from the corresponding author upon request. All the raw sequencing reads that support the findings of this study and its supplementary information file have been deposited in the in the National Center for Biotechnology Information Sequence Read Archive (SRA) and are accessible through BioProject ID PRJNA281110 [https://www.ncbi.nlm.nih.gov/bioproject/?term=PRJNA281110]. All the information of SRA, including project number, total base pairs, and name of accession are list in Supplementary Table 2. The source data underlying Fig. 5 are provided as a Source Data file.

## Source data

Source Data

## Code availability

All custom scripts are available at Github (https://github.com/avinashkarn/analyze_amplicon) or at Bitbucket (https://bitbucket.org/cornell_bioinformatics/amplicon).
