# Supplementary material for: Haplotyping the Vitis collinear core genome with rhAmpSeq improves marker transferability in a diverse genus
Source: Nat Commun. 2020 Jan 21;11:413. doi: 10.1038/s41467-019-14280-1 (PMC6972940; doi:10.1038/s41467-019-14280-1)
Supplement: Supplementary file 1 — Supplementary Information [file 41467_2019_14280_MOESM1_ESM.pdf]

Haplotyping the *Vitis* collinear core genome with rhAmpSeq improves marker transferability in a diverse genus

Zou *et al*.

Supplementary Fig. 1

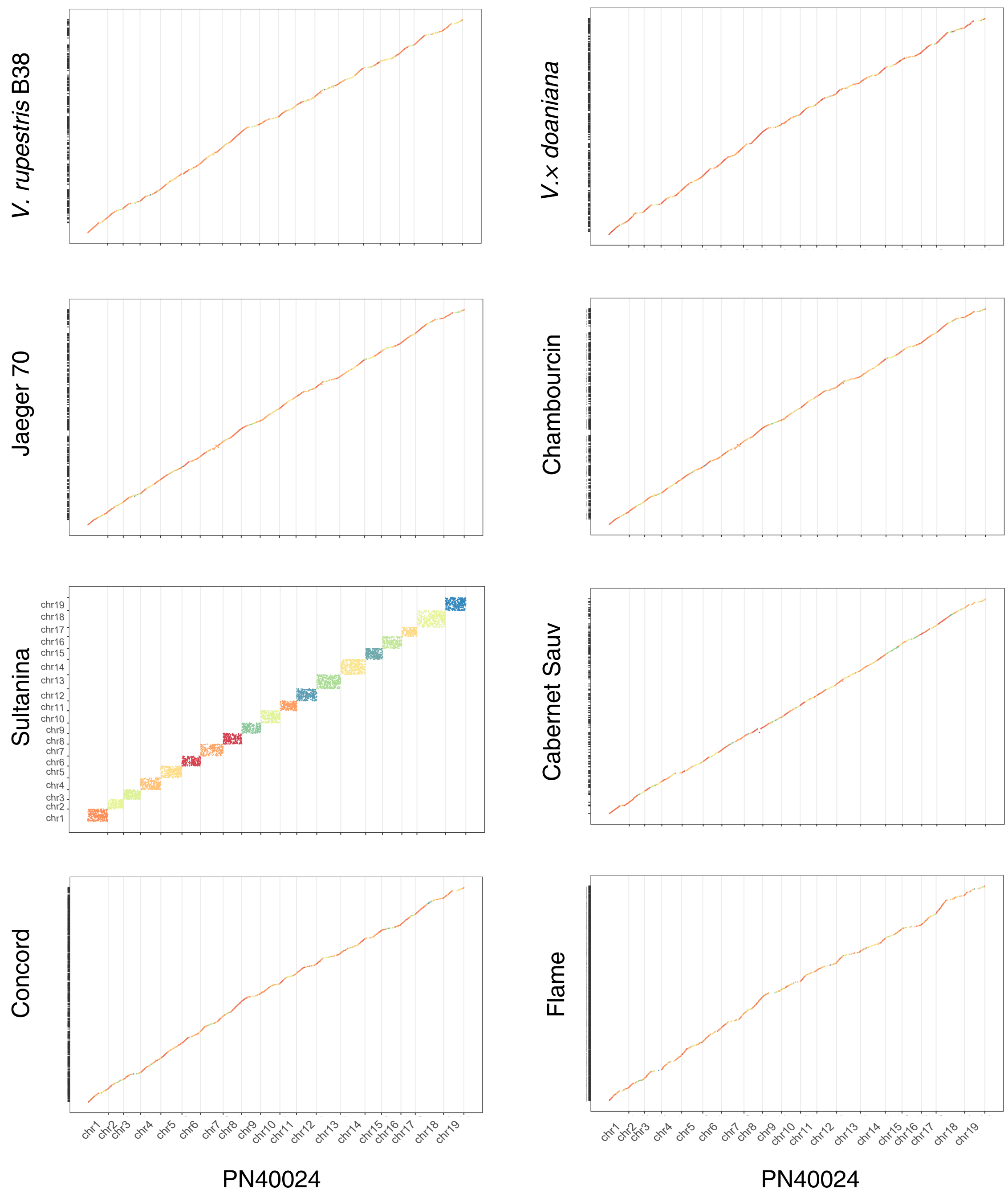

Supplementary Figure 1. Dot plot depicting the collinear one-to-one alignments between individual assemblies and the reference genome PN40024. Only alignments that are longer than 5000bp and the contigs are greater than 50000bp are presented. The arrangement of contigs within each chromosome is not ordered in Sultanina assembly.

Supplementary Fig. 2

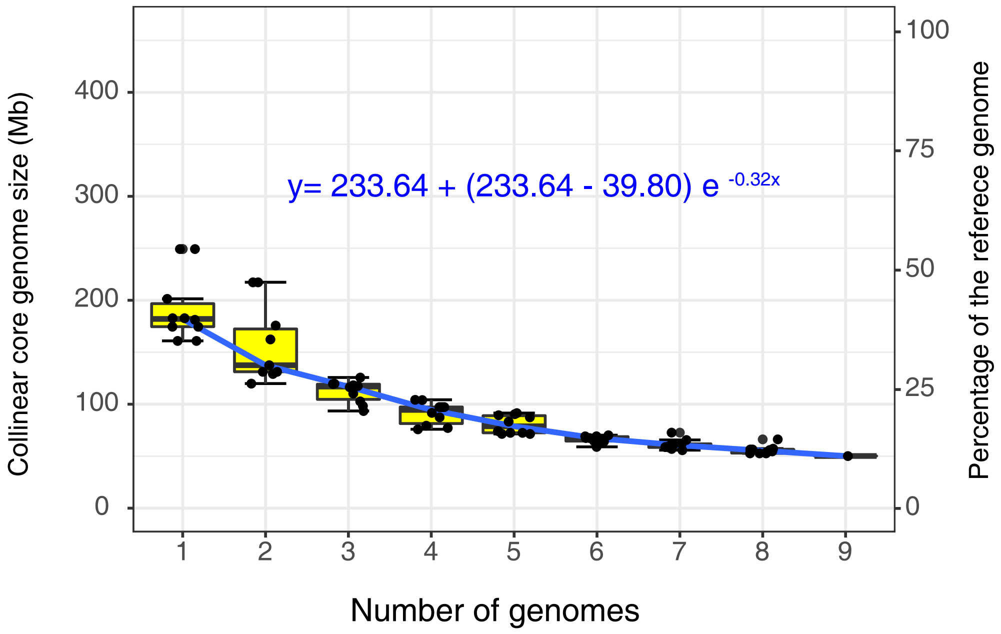

Supplementary Figure 2. The total length of the collinear core genome decreases as an exponential decay as more assembled genomes are included in the core genome construction. Ten simulations were performed using different numbers of assembled genomes (x-axis) to calculate the absolute collinear core genome size (left y-axis) and the percentage of the reference genome (right y-axis). The blue line shows the fitted expo-nential decay function by the Self-Starting Nls Asymptotic Regression Model. The core genome size reaches a plateau at 39.80Mb with 17 assemblies in the model.

Supplementary Fig. 3

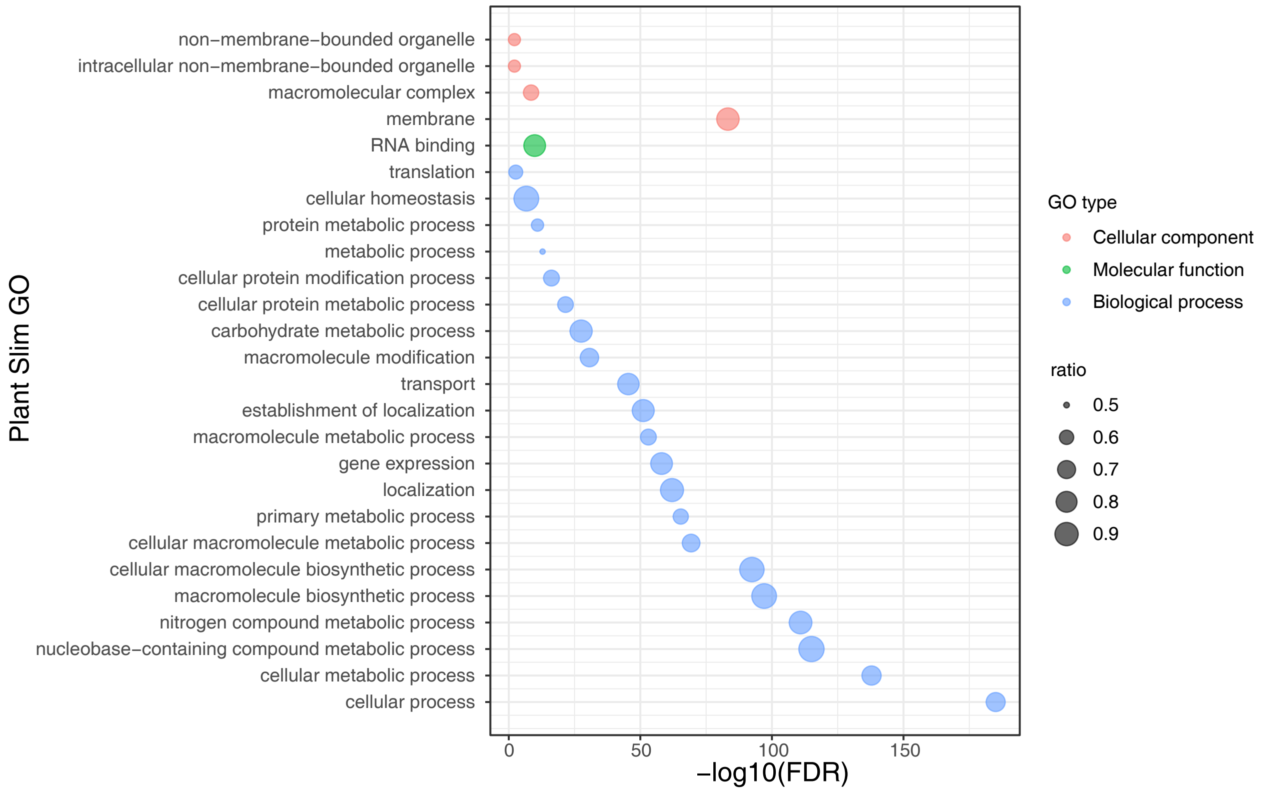

Supplementary Figure 3. Enriched gene ontology categories in core genes are shown in bubble plot. The bubble size represents the proportion of core genes in this functional category, the dot color represents the types of GO annotation. The x-axis is the base-10 logarithm of FDR.

Supplementary Fig. 4

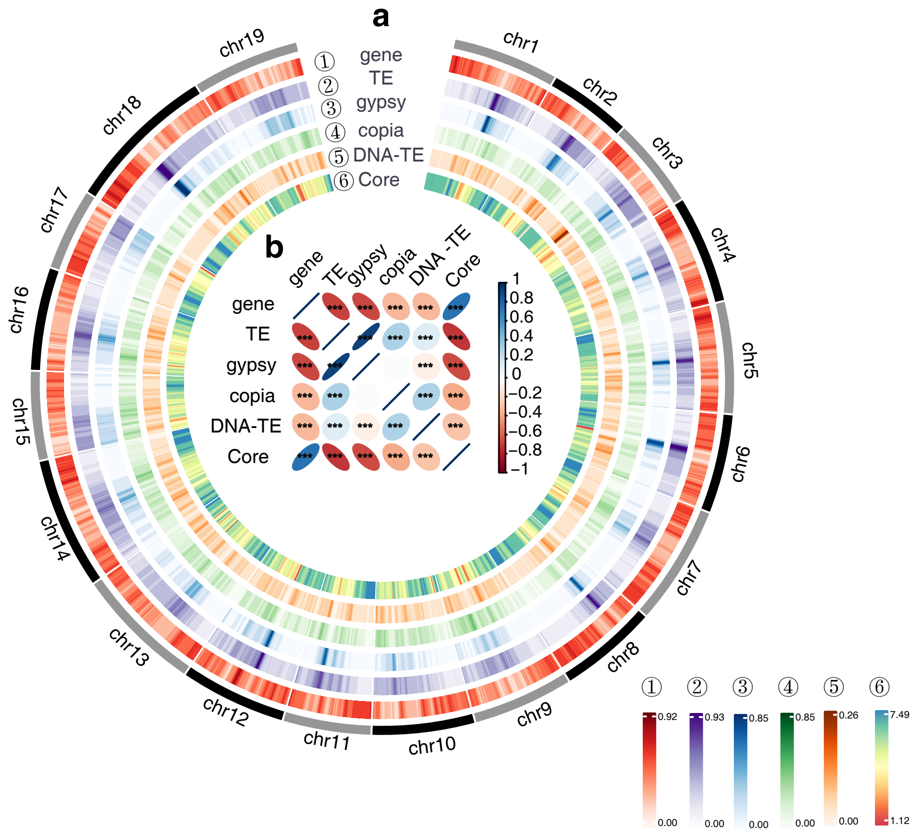

Supplementary Figure 4. Circos plot depicting the correlation between the core genome and other genome features. a. Circos plot depicting coverage of the genes, transposable elements (TE), different types of transposons (Gypsy, Copia, DNA-TE) and the core genome. The core genome was constructed based on the reference genome plus the nine assemblies. For each track, the coverage for this feature was calculated for each sliding window on the reference genome (length 1Mbp, step size 100 kbp). **b.** Spearman rank correlation coefficient between each pairs of genome features and the core genome are shown. *: P-value <0.05, **: P-value<0.01, ***: P-value <0.0001

Supplementary Fig. 5

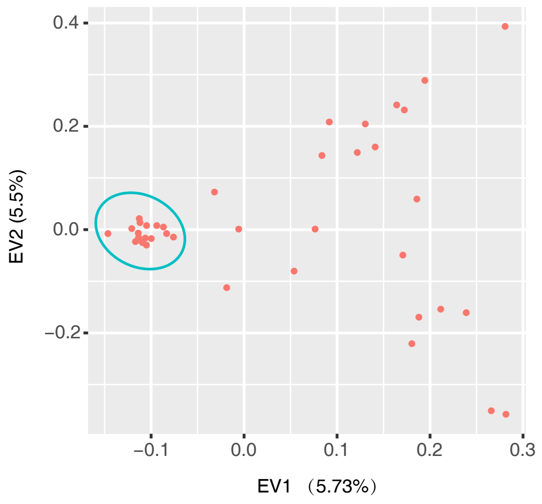

Supplementary Figure 5. Principal Component Analysis (PCA) representing the genetic diversity of the samples in the diversity panel. PCA plots of the first two eigenvectors of all 40 accessions we examined. Individuals in the blue circle are from *Vitis vinifera.*

Supplementary Fig. 6

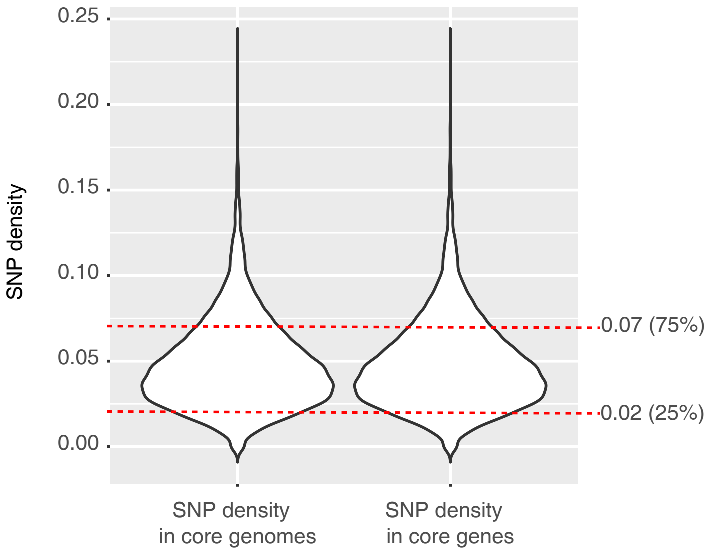

Supplementary Figure 6. Distribution of the SNP density in the core genome and core genes. Violin plots illustrate the kernel probability density of SNP density (y axis).

Supplementary Fig. 7

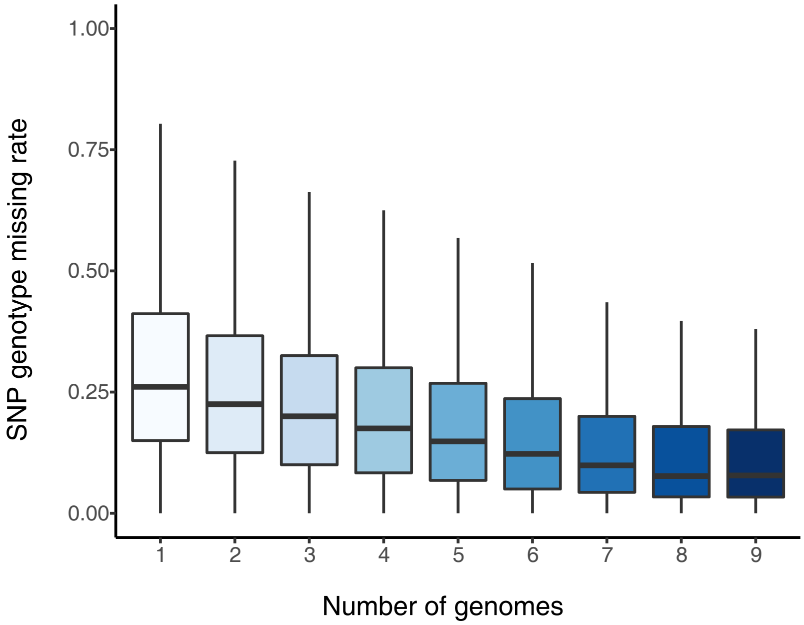

Supplementary Figure 7. The genotype missing rate in the *Vitis* diversity panel decreases as an exponential decay as more assembled genomes are included in the core genome construction. Ten simulations were performed using different numbers of assembled genomes (x-axis) to calculate the SNP genotype missing rate (y-axis). As the true core genome is approached, the missing rate should converge on an asymptote and variance should reach a minimum, which are non-zero due to technical error in resequencing.

Supplementary Fig. 8

a

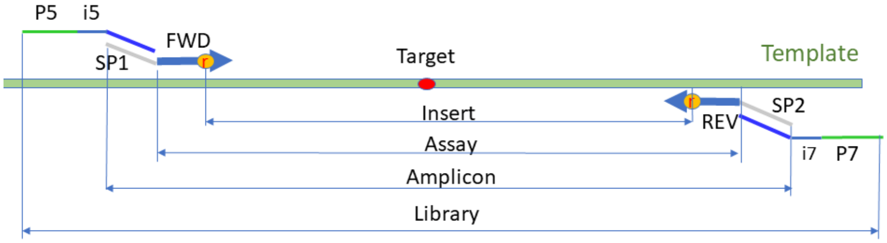

Assay description

	Description
Template	Regions of DNA for rhAmpSeq assay design
Target	Target region for assay design
FWD	rhAmpSeq target-specific foward primer
REV	rhAmpSeq target-specific reverse primer
SP1	adaptor1 sequence
SP2	adaptor2 sequence
Insert	Amplification product not including primers and adaptors
Assay	Amplification product including primers but not adaptors
Amplicon	Amplification product after PCR1
Library	Amplification product after PCR2
r	RNA-base–containing blocker

b

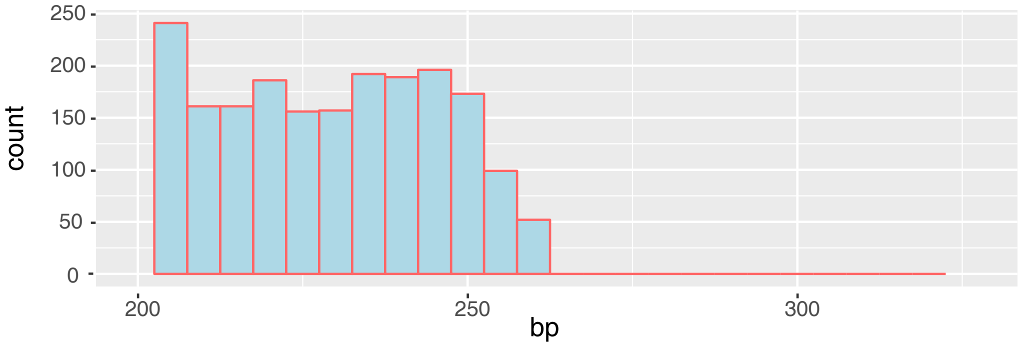

c

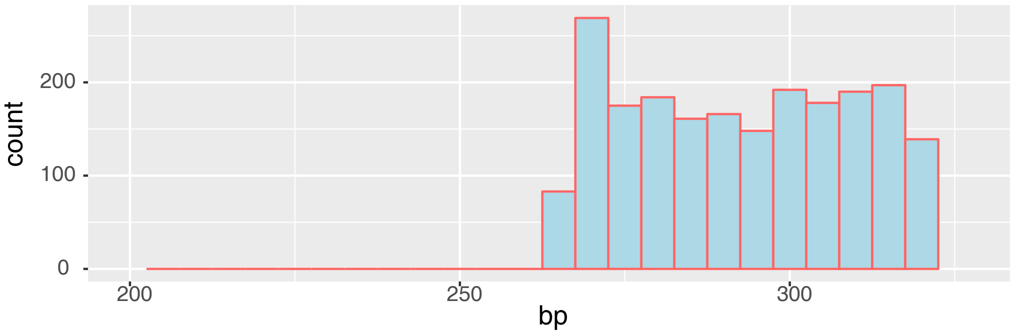

Supplementary Figure 8. Components of a rhAmpSeq assay and the length distributions of 2000 rhAmpSeq targets and amplicons. a. Components of a rhAmpSeq assay. **b.** Histogram of target assay size. Target assays contain the forward primer, reverse primer and insert, but exclude the Illumina adapter sequences (sp1 and sp2). **c.** Histogram of amplicon size. The amplicon is defined as the target assay plus the Illumina sequencer adapters, and is the PCR product after the second round of amplification.

Supplementary Fig. 9

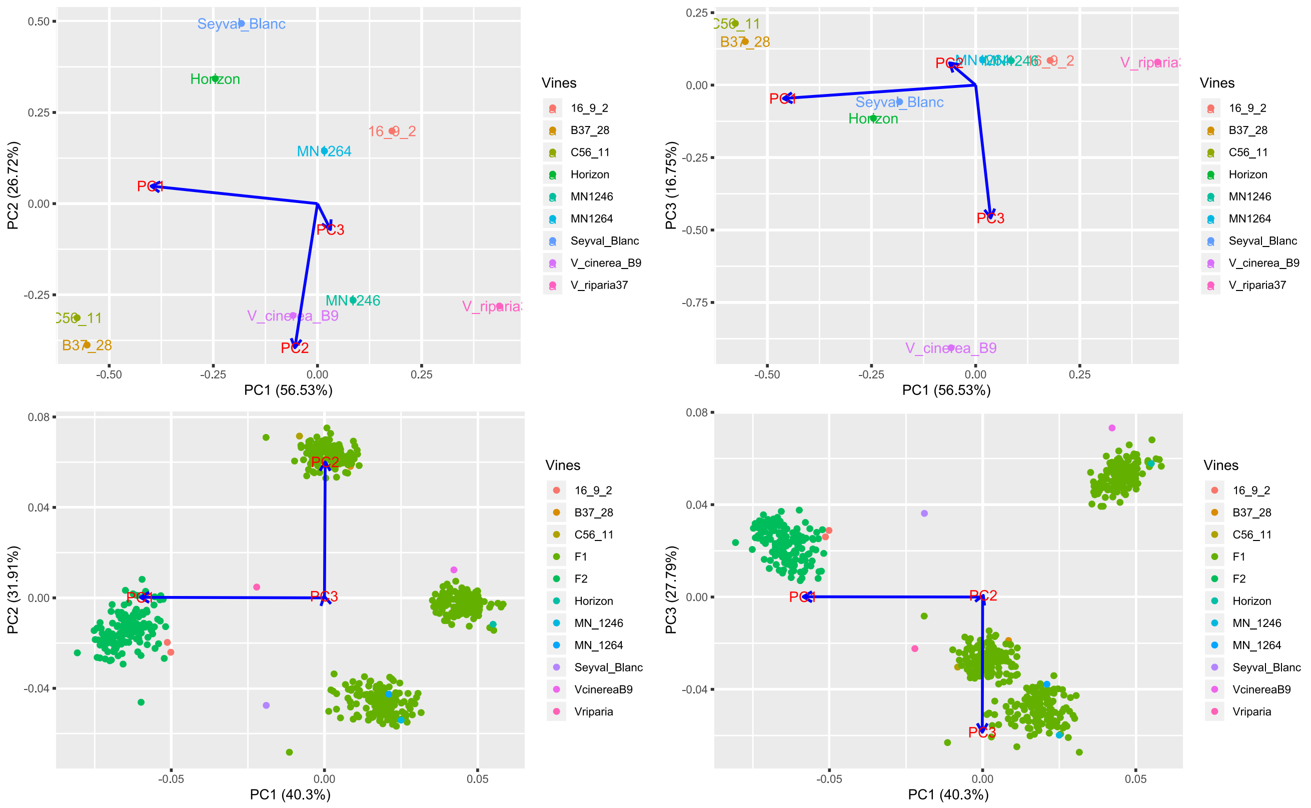

Supplementary Figure 9. Multidimensional scaling (MDS) plot depicting the genetic diversity in the parents and progeny of four families studied here. Multidimensional scaling (MDS) plot of the top three principal coordinates (PCs) including only the parents of the four families (top), and all parents and progeny (bottom).

Supplementary Fig. 10

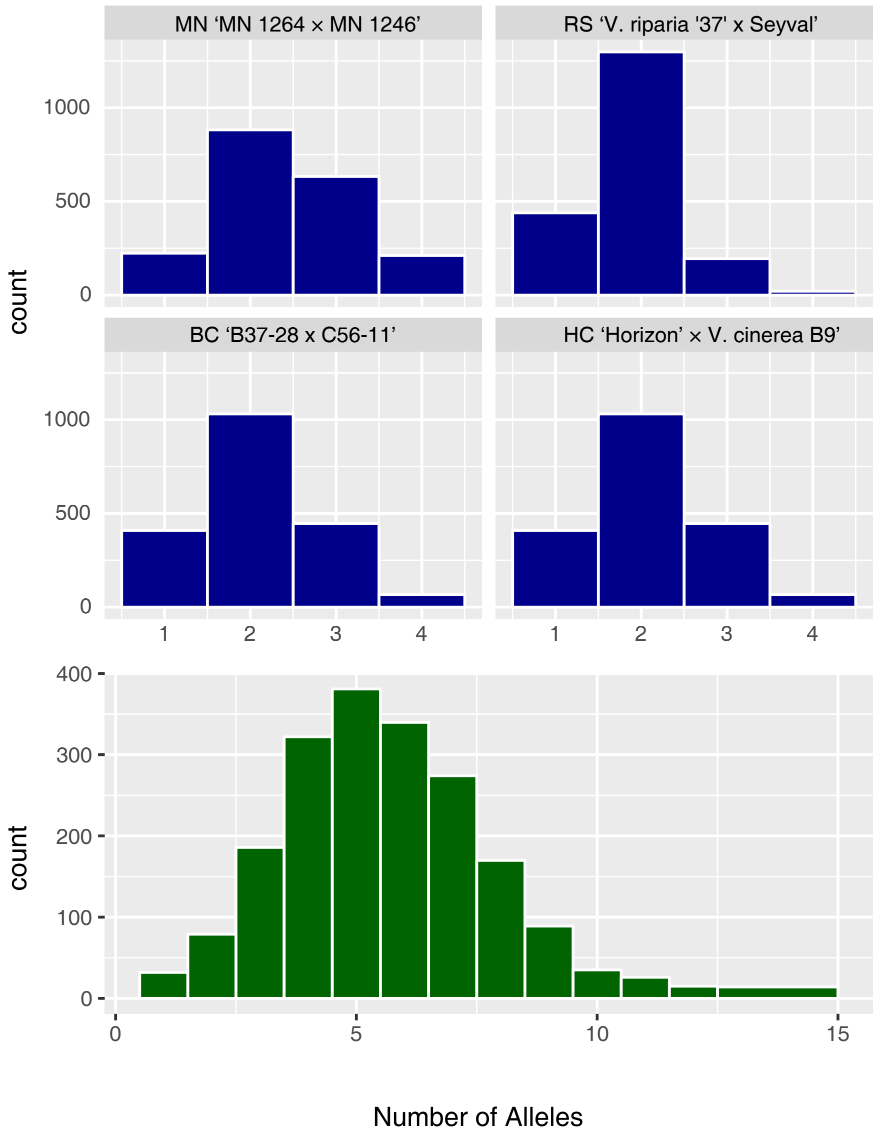

Supplementary Figure 10. Number of haplotype alleles in each population and overall. For each population, the number of markers returning 1 to 4 alleles is shown.The bottom histogram shows the total number of alleles observed across all 4 populations. The theoretical maximum number of alleles is 14 across all four families

Supplementary Fig. 11

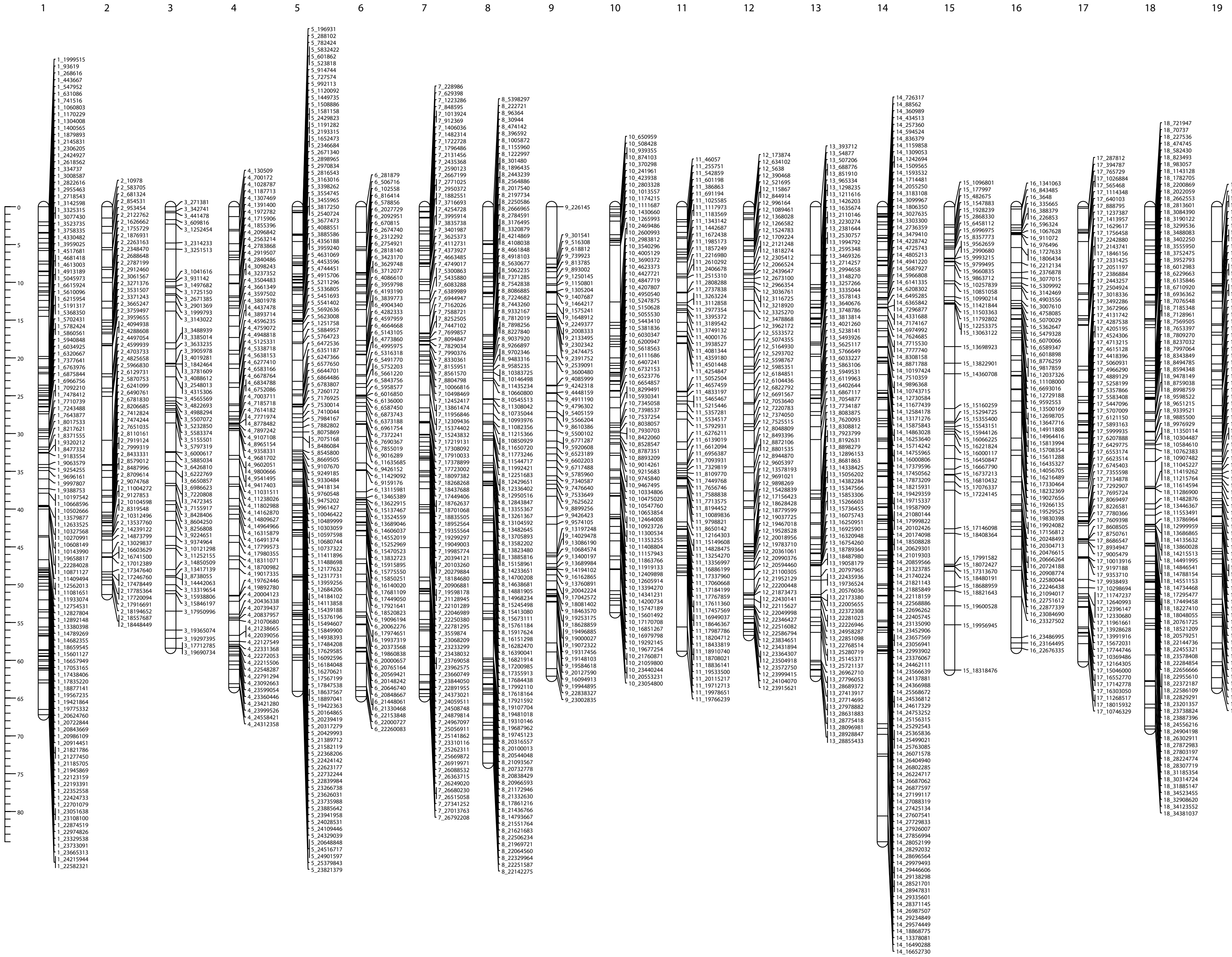

Supplementary Figure 11. Consensus genetic map derived from four mapping populations. The linkage map consists of 1,661 markers with total map size of 1198.1 cM. The 19 chromosomes are the labeled above each linkage group. Genetic position (cM) of each marker is on the scale on the left-hand side of the plot, and physical positions for each marker are listed as ‘chromosome_physical position’.

Supplementary Table 1. Summary statistics of genome assemblies and collinear alignment

Origin	Accession	USDA ID	Assembly	Scaffold N50	10x statistics			Total bases	Total bases of chained alignments(Mbp)*	Total bases of collinear alignments (Mbp)**	Total bases of overlapped with CDS before filtering (Mbp)	Total bases of overlapped with CDS after filtering (Mbp)
			Method	(kbp)	Raw cov	Effective Cov	Molecular Len	(Mbp)				
Wild species	***Vitis cinerea* B9**	PI_588154	10x	364	48.15	39.31	21.57	415	333.3	181	27.6	26.0
	***Vitis rupestris* B38**	PI_588160	10x	545	65.89	46.73	57.76	524	350.8	160	26.1	23.9
Hybrid of wild species	***Vitis x doaniana (V. acerifolia x V. mustangensis)***	PI_588149	10x	1767	66.34	47.06	65.74	472	378.6	174	27.2	25.1
	**Jaeger 70 (*V. aestivalis lincecumii* x *V. rupestris*)**	PI_588234	10x	292	31.60	22.40	64.44	503		161	26.6	23.7
Hybrid of wild species and cultivars	***Vitis* sp. cv. Chambourcin**	PI_588075	10x	538	64.83	46.98	56.63	523	361.2	202	28.3	25.4
	***Vitis* sp. cv. Concord**	PI_588077	10x	324	49.22	39.81	37.66	491	305.4	183	27.1	22.6
Domesticated	***Vitis vinifera* cv. Sultanina**	NA	multiple	278	NA	NA	NA	544	484.3	278	31.5	24.8
	***Vitis vinifera* cv. Cabernet Sauvignon**	NA	PacBio	2173	NA	NA	NA	591	399.1	218	26.9	23.7
	***Vitis vinifera* cv. Flame Seedless**	NA	10x	1980	47.02	38.07	74.08	543	404.3	250	26.7	16.1
Reference genome	***Vitis vinifera* cv PN40024**	NA	multiple	chromosome	NA	NA	NA	458				

* the total length of collinear alignment including gaps within the chains

**the total length of collinear alignment without gaps

Supplementary Table 2. Summary statistics of samples used for estimating genetic polymorphism in the *Vitis* genus

Sample Accession	Included?	BioProject	Depth (Mbp)	Taxon	Sample_Name	cultivar
SRR5626750	Y	PRJNA387534	30440	Vitis vinifera	Nebbiolo_CVT423	Nebbiolo
SRR5627784	N	PRJNA388292	4205	Vitis vinifera subsp. sylvestris	Wild grape_Armenia(Vitis vinifera ssp. sylvestris)	
SRR5627786	Y	PRJNA388292	9295	Vitis vinifera subsp. sylvestris	Wild grape_Turkmenistan2(Vitis vinifera ssp. sylvestris)	
SRR5627787	Y	PRJNA388292	8569	Vitis vinifera subsp. sylvestris	Wild grape_Azerbaijan1(Vitis vinifera ssp. sylvestris)	
SRR863618	N	PRJNA203534	45460	Vitis vinifera	tannat genomic reads	Tannat
SRR5627781	Y	PRJNA388292	8754	Vitis vinifera	Grape_Muscat of Alexandria(Vitis viniferassp. Vinifera)	Muscat of Alexandria
SRR5627785	Y	PRJNA388292	11469	Vitis vinifera subsp. sylvestris	Wild grape_Pakistan1(Vitis vinifera ssp. sylvestris)	
SRR5627789	N	PRJNA388292	9300	Vitis vinifera subsp. sylvestris	Wild grape_Georgia(Vitis vinifera ssp. sylvestris)	
SRR863595	Y	PRJNA203534	16721	Vitis vinifera	tannat genomic reads	Tannat
SRR769824	N	PRJNA192798	1072	Vitis vinifera	Vitis vinifera cv. Italia	Italia
SRR2015301	N	PRJNA283439	24190	Vitis vinifera	genome resequencing of Vitis vinifera cv. 'Riesling'	
SRR5626393	Y	PRJNA387534	19247	Vitis vinifera	Nebbiolo_CVT185	Nebbiolo
SRR354201	Y	SRP009057	7945	Vitis vinifera	Red_Globe	
SRR769842	N	PRJNA192798	2558	Vitis amurensis	W6	
SRR769843	Y	PRJNA192798	2657	Vitis riparia	W7	
SRR769840	Y	PRJNA192798	2906	Vitis cinerea	W4	
SRR2015347	Y	PRJNA283439	8261	Vitis girdiana	genome resequencing of Vitis girdiana	
SRR769844	Y	PRJNA192798	2497	Vitis amurensis	W8	
SRR5627783	Y	PRJNA388292	8895	Vitis vinifera subsp. sylvestris	Wild grape_Turkmenistan1(Vitis vinifera ssp. sylvestris)	
SRR2015348	Y	PRJNA283439	5917	Vitis palmata	genome resequencing of Vitis palmata	
SRR5712111	Y	PRJNA390884	9768	Vitis vinifera	Kishmish Vatkana	Kishmish vatkana
SRR5626056	Y	PRJNA387534	32686	Vitis vinifera	Nebbiolo_CVT71	Nebbiolo
SRR5627799	N	PRJNA388292	30858	Vitis vinifera	Grape_Chardonnay(Vitis viniferassp. Vinifera)	Chardonnay
SRR5627798	Y	PRJNA388292	7553	Vitis vinifera	Grape_Gamay Noir(Vitis viniferassp. Vinifera)	Gamay Noir3
SRR5627793	N	PRJNA388292	8214	Vitis vinifera	Grape_Semillion(Vitis viniferassp. Vinifera)	Semillion12
SRR5627792	N	PRJNA388292	13108	Vitis vinifera subsp. sylvestris	Wild grape_Pakistan2(Vitis vinifera ssp. sylvestris)	
SRR5627791	N	PRJNA388292	12078	Vitis vinifera subsp. sylvestris	Wild grape_Pakistan3(Vitis vinifera ssp. sylvestris)	
SRR5627790	Y	PRJNA388292	8779	Vitis vinifera subsp. sylvestris	Wild grape_Azerbaijan2(Vitis vinifera ssp. sylvestris)	
SRR5483432	Y	PRJNA384014	16844	Vitis amurensis	Leaves of Vitis amurensis	zuoshaner
SRR5483433	Y	PRJNA384014	16822	Vitis amurensis	Leaves of hybrid of Vitis amurensis and Vitis vinifera	Zuoyouhong
SRR5627794	N	PRJNA388292	8468	Vitis vinifera	Grape_Riesling(Vitis viniferassp. Vinifera)	Riesling 4
SRR769832	Y	PRJNA192798	2837	Vitis amurensis	D	
SRR769841	Y	PRJNA192798	2427	Vitis coignetiae	W5	
SRR769837	Y	PRJNA192798	2295	Vitis davidii	W1	
SRR2016902	Y	PRJNA283439	7887	Vitis riparia x Vitis rupestris	genome resequencing of Vitis hybrid 'C3309'	
SRR769839	Y	PRJNA192798	2881	Vitis aestivalis	W3	
SRR769838	Y	PRJNA192798	2836	Vitis thunbergii	W2	
SRR5803838	N	PRJNA392287	12242	Vitis vinifera	Vermentino	Vermentino
SRR5803839	N	PRJNA392287	7472	Vitis vinifera	Carignano	Carignano
SRR5803836	N	PRJNA392287	19956	Vitis vinifera	Cannonau	Cannonau
SRR354199	Y	SRP009057	6177	Vitis vinifera	Autumn_Royal	
SRR5627796	Y	PRJNA388292	17314	Vitis vinifera	Grape_Primitivo(Vitis viniferassp. Vinifera)	Primitivo03
SRR354198	Y	SRP009057	8186	Vitis vinifera	Italia	
SRR5803837	N	PRJNA392287	4278	Vitis vinifera	Bovale Sardo	Bovale Sardo
SRR5627802	Y	PRJNA388292	6948	Vitis vinifera	Grape_Traminer(Vitis viniferassp. Vinifera)	Traminer 1
SRR5627801	Y	PRJNA388292	23482	Vitis vinifera	Grape_Zinfindel(Vitis viniferassp. Vinifera)	Zinfandel 03
SRR5627800	Y	PRJNA388292	10619	Vitis vinifera	Grape_Aramon(Vitis viniferassp. Vinifera)	Aramon
Vitis romanetii C-166-026	Y		75048	Vitis romanetii	C-166-026	
Vitis rupestris Pillans	Y		81248	Vitis rupestris	Pillans	Pillans
MN1264	Y		15644	Vitis interspecific hybrid	MN1264	MN1264
MN1246	Y		21731	Vitis interspecific hybrid	MN1246	MN1246
Horizon	Y		4532	Vitis interspecific hybrid	Horizon	Horizon
Illinois 547-1	Y		3810	Vitis interspecific hybrid	Illinois 547-1	Illinois 547-1
Vitis rupestris B38	Y		34211	Vitis rupestris	B38	B38
