# Supplementary material for: Haplotyping the Vitis collinear core genome with rhAmpSeq improves marker transferability in a diverse genus
Source: Nat Commun. 2020 Jan 21;11:413. doi: 10.1038/s41467-019-14280-1 (PMC6972940; doi:10.1038/s41467-019-14280-1)
Supplement: Supplementary file 2 — Reporting Summary [file 41467_2019_14280_MOESM2_ESM.pdf]

nature research

Corresponding author(s): Lance Cadle-Davidson

Last updated by author(s): Dec 12, 2019

Reporting Summary

Nature Research wishes to improve the reproducibility of the work that we publish. This form provides structure for consistency and transparency in reporting. For further information on Nature Research policies, see Authors & Referees and the Editorial Policy Checklist.

Statistics

For all statistical analyses, confirm that the following items are present in the figure legend, table legend, main text, or Methods section.

n/a	Confirmed	
☐	☒	The exact sample size (*n*) for each experimental group/condition, given as a discrete number and unit of measurement
☒	☐	A statement on whether measurements were taken from distinct samples or whether the same sample was measured repeatedly
☐	☒	The statistical test(s) used AND whether they are one- or two-sided<br>*Only common tests should be described solely by name; describe more complex techniques in the Methods section.*
☒	☐	A description of all covariates tested
☐	☒	A description of any assumptions or corrections, such as tests of normality and adjustment for multiple comparisons
☐	☒	A full description of the statistical parameters including central tendency (e.g. means) or other basic estimates (e.g. regression coefficient) AND variation (e.g. standard deviation) or associated estimates of uncertainty (e.g. confidence intervals)
☐	☒	For null hypothesis testing, the test statistic (e.g. *F*, *t*, *r*) with confidence intervals, effect sizes, degrees of freedom and *P* value noted<br>*Give P values as exact values whenever suitable.*
☒	☐	For Bayesian analysis, information on the choice of priors and Markov chain Monte Carlo settings
☒	☐	For hierarchical and complex designs, identification of the appropriate level for tests and full reporting of outcomes
☐	☒	Estimates of effect sizes (e.g. Cohen's *d*, Pearson's *r*), indicating how they were calculated

Our web collection on statistics for biologists contains articles on many of the points above.

Software and code

Policy information about availability of computer code

Data collection	No software was used for data collection
Data analysis	The open source software we used is described in the method section. Custom code for rhAmpSeq analysis has been deposited in the Github and Bitbucket listed in the manuscript.

For manuscripts utilizing custom algorithms or software that are central to the research but not yet described in published literature, software must be made available to editors/reviewers. We strongly encourage code deposition in a community repository (e.g. GitHub). See the Nature Research guidelines for submitting code & software for further information.

Data

Policy information about availability of data

All manuscripts must include a data availability statement. This statement should provide the following information, where applicable:

- Accession codes, unique identifiers, or web links for publicly available datasets
- A list of figures that have associated raw data
- A description of any restrictions on data availability

Raw sequencing data that generated in this study have been deposited in the NCBI Sequence Read Archive (SRA) under project PRJNA281110, to be released upon publication. The genome assemblies will be deposited at NCBI before publication, and accession numbers will be provided in the final manuscript. Polymorphism data will be deposited in CyVerse Data Common with a link to the location provided in the final manuscript.

Field-specific reporting

Please select the one below that is the best fit for your research. If you are not sure, read the appropriate sections before making your selection.

☒ Life sciences ☐ Behavioural & social sciences ☐ Ecological, evolutionary & environmental sciences

For a reference copy of the document with all sections, see nature.com/documents/nr-reporting-summary-flat.pdf

Life sciences study design

All studies must disclose on these points even when the disclosure is negative.

Sample size	Ten genome assemblies were used in the core genome construction and 40 samples were used to assess polymorphism. The marker system we developed was tested in four families. The rationale of why these samples were chosen and why they were sufficient is outlined in the manuscript.
Data exclusions	We excluded some Vitis vinifera resequencing samples to achieve a balanced genetic representation in the data as we described in the manuscript.
Replication	The marker system we developed was tested in four independent families, as a stronger form of replication than repeated genotyping.
Randomization	Seedlings are randomly planted and genotyped, which is the only relevant randomization.
Blinding	The investigators were blind to the phenotypic data.

Reporting for specific materials, systems and methods

We require information from authors about some types of materials, experimental systems and methods used in many studies. Here, indicate whether each material, system or method listed is relevant to your study. If you are not sure if a list item applies to your research, read the appropriate section before selecting a response.

Materials & experimental systems

n/a	Involved in the study	
☒	☐	Antibodies
☒	☐	Eukaryotic cell lines
☒	☐	Palaeontology
☒	☐	Animals and other organisms
☒	☐	Human research participants
☒	☐	Clinical data

Methods

n/a	Involved in the study	
☒	☐	ChIP-seq
☒	☐	Flow cytometry
☒	☐	MRI-based neuroimaging
