# Supplementary material for: Haplotyping the Vitis collinear core genome with rhAmpSeq improves marker transferability in a diverse genus
Source: Nat Commun. 2020 Jan 21;11:413. doi: 10.1038/s41467-019-14280-1 (PMC6972940; doi:10.1038/s41467-019-14280-1)
Supplement: Supplementary file 3 — Description of Additional Supplementary Files [file 41467_2019_14280_MOESM3_ESM.docx]

**Description of Additional Supplementary Files**

File name: Supplementary Data 1
Description: The sequences of the 2000 rhAmpSeq primer pairs tested in the four mapping families. /rhSeq-f/ and /rhSeq-r/ indicate 5' Illumina linker sequences. An RNA nucleotide is indicated by r, and 3' blockers are indicated by /GT#/. The four columns labeled “KeyFile” indicate the information provided for the pipeline analyze_amplicon.pl for haplotype allele calling.

File name: Supplementary Data 2
Description: Summary of the genetic maps for the four families, including correlation between the genetic order of the markers to their physical positions (PN40024 12X.2) and coverage of the physical genome.
